# BK Channels Control Cerebellar Purkinje and Golgi Cell Rhythmicity In Vivo

**DOI:** 10.1371/journal.pone.0007991

**Published:** 2009-11-24

**Authors:** Guy Cheron, Matthias Sausbier, Ulrike Sausbier, Winfried Neuhuber, Peter Ruth, Bernard Dan, Laurent Servais

**Affiliations:** 1 Laboratory of Electrophysiology, Université Mons-Hainaut (UMH), Mons, Belgium; 2 Laboratory of Neurophysiology and Movement Biomechanics, Université Libre de Bruxelles (ULB), Brussels, Belgium; 3 Department of Pharmacology and Toxicology, Institute of Pharmacy, Universität Tübingen, Tübingen, Germany; 4 Institute of Anatomy, University of Erlangen-Nürnberg, Erlangen, Germany; 5 Department of Neurology, Hôpital Universitaire des Enfants Reine Fabiola, Université Libre de Bruxelles (ULB), Brussels, Belgium; 6 Department of child neurology, Hôpital Robert Debré, Paris, France; University of Maryland School of Pharmacy, United States of America

## Abstract

Calcium signaling plays a central role in normal CNS functioning and dysfunction. As cerebellar Purkinje cells express the major regulatory elements of calcium control and represent the sole integrative output of the cerebellar cortex, changes in neural activity- and calcium-mediated membrane properties of these cells are expected to provide important insights into both intrinsic and network physiology of the cerebellum. We studied the electrophysiological behavior of Purkinje cells in genetically engineered alert mice that do not express BK calcium-activated potassium channels and in wild-type mice with pharmacological BK inactivation. We confirmed BK expression in Purkinje cells and also demonstrated it in Golgi cells. We demonstrated that either genetic or pharmacological BK inactivation leads to ataxia and to the emergence of a beta oscillatory field potential in the cerebellar cortex. This oscillation is correlated with enhanced rhythmicity and synchronicity of both Purkinje and Golgi cells. We hypothesize that the temporal coding modification of the spike firing of both Purkinje and Golgi cells leads to the pharmacologically or genetically induced ataxia.

## Introduction

Since Purkinje cells (PCs) are solely responsible for the output of the cerebellar cortex, regulation of their firing is central for motor coordination. This regulation does not only depend on synaptic input but also on intrinsic excitability. PCs spontaneously fire simple spikes in tonic, bursting, or silent modes both in *vivo* and *in vitro*. This intrinsic excitability is driven by resurgent Na^+^ channels [1], voltage-gated Ca^2+^ channels, and Ca^2+^-activated K^+^ channels [2], [3]. The latter are categorized as small (SK) or large (BK) conductance channels. Maximal activation of BK channels (alias maxiK, KCNMA1, K_Ca_1.1 or slowpoke) requires both membrane depolarization and increased intracellular Ca^2+^ [4], [5]. Various functions have been suggested for BK channels [6], including climbing fiber response modulation [2]. It is thought that the massive Ca^2+^ entry that follows activation by the climbing fiber causes an outward K^+^ current that hyperpolarizes the cell thereby preventing simple spike firing for ∼20 ms *in vivo*. *In vitro* BK channel blockade leads to a slight simple spike firing rate increase if applied during tonic firing [2], [7] and to a complex modification of burst pattern if applied during bursting period [3], [7], [8]. This suggests that BK channels are critical for the fine regulation of Purkinje cells' intrinsic excitability.

To further understand how BK channels affect PC firing properties, and thus cerebellar function, mice deficient in the BK channel (BK^−/−^) have been generated [7]. In slice preparations, PCs of BK^−/−^ mice have a dramatic decrease in spontaneous firing relative to PCs from wild-type (WT) mice, partially explained by an increase in time in silent mode. In addition, the paired-pulse depression at the PC deep cerebellar neurons is increased. These findings led to the hypothesis that the major motor coordination impairment observed in BK^−/−^ mice results from a decreased net inhibition of deep cerebellar nuclei by the PCs [7].

To test this hypothesis, we recorded the spontaneous and stimulus-evoked activities of PCs in alert BK^−/−^ mice and WT controls. We found that PC activity was only mildly decreased in BK^−/−^ mice relative to WT, but that their cerebellum presented a beta rhythm local field potential oscillation phase-locked with ultra-rhythmic Purkinje and Golgi cells. We demonstrated the existence of BK channels in Golgi cells, which could partly explain the phase-locking of these cells to the abnormal beta rhythm in BK^−/−^ mice. We also demonstrated that this PC firing pattern and the ataxic behavior of BK^−/−^ mice were reproduced *in vivo* by microinjection of a BK channel blocker into the vermis of WT mice.

## Methods

### Mice

Eight to 12 week-old male and female BK^−/−^ mice and their WT littermates, generated as described previously [7], were used in all experiments. This study was conducted with the permission of the University of Mons Ethics Committee and was in agreement with International Guidelines. A first set of 10 mice (five WT and five BK^−/−^) was examined by an investigator blind to the genotype to characterize the differences between Purkinje and Golgi cells firing in BK^−/−^ and WT mice. Then a second set of fourteen identified BK^−/−^ mice was investigated in order to further study the characteristics of the local field potential and neuronal discharge patterns. Finally, a set of ten WT mice was used to study the effect of paxilline microinjection.

### Surgical Preparation

Mice were anesthetized with xylido-dihydrothiazin (Rompun®, Bayer, 10 mg/kg) and ketamine (Ketalar®, Pfizer, 100 mg/kg). Animals were administered an additional dose of xylido-dihydrothiazin (3 mg/kg) and ketamine (30 mg/kg) if they presented agitation or markedly increased respiration or heart rate during the procedure. In addition, local anesthesia (0.5 mL of 20 mg/mL lidocaine and adrenaline [1∶80000, Xylocaine®, Astra Zeneca]) was administered subcutaneously during soft tissue removal. Two small bolts were cemented to the skull to immobilize the head during the recording sessions and a silver reference electrode was placed on the surface of the parietal cortex. An acrylic recording chamber was constructed around a posterior craniostomy, covered by a thin layer of bone wax (Ethicon®, Johnson & Johnson) before the recording sessions. Twenty-four hours after anesthesia, alert mice were immobilized for the recording session. The dura mater was removed locally above the vermis. Recordings were performed in lobules IV–VIII and the location of the electrodes (depth and lobule) was noted. To avoid useless stress for the animals and movement artefacts, recordings were performed in a quiet room and only when animals were calm in the setting.

### Single-Unit Recordings

**Figure 1:**
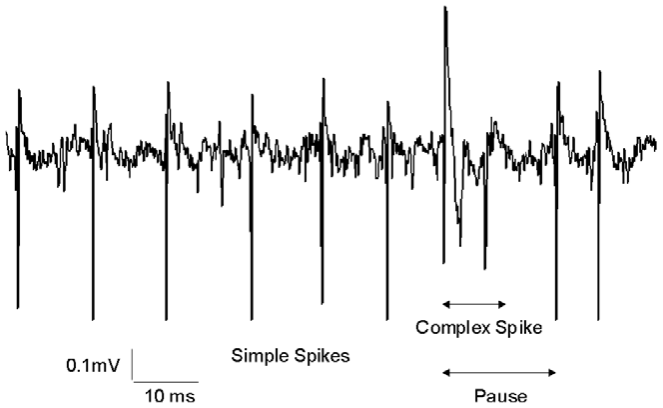
Extracellular recording of a Purkinje cell in an alert mouse. Note the two types of spikes, and the pause induced by the complex spike in the simple spike train.

Single-unit recordings were performed with glass micropipettes filled with NaCl 0.2 M (1.5–5 MΩ impedance). A neural signal was considered as originating from a PC if it presented two types of spiking activities: simple spikes characterized by single depolarization (300–800 µs) occurring between 20 and 200Hz and complex spikes characterized by an initial fast depolarization (300–600 µs) followed by smaller and relatively constant wavelets. It was considered that simple and complex spikes originated from the same PC when a transient pause (∼15 ms) in simple spike firing followed each complex spike (Fig. 1). Golgi cells were identified according to their firing properties [9], [10], namely a slow (4–20 Hz) and irregular discharge with interspike intervals always longer than 50 ms. Recordings were analyzed if longer than 60 seconds.

### Multi-Unit Recordings

Multiple recordings along the same parallel fiber axis were performed by means of seven linearly arranged, quartz-insulated, platinum-tungsten fiber-microelectrodes (outer and shaft diameter of 80 µm and 25 µm, respectively) with 250-µm inter-electrode spacing. Each microelectrode was mounted into a stretched elastic rubber tube enabling proper positioning via DC-micromotors (resolution of 0.27 µm) [11].

### Microinjections

Carbenoxolone (48 mM) diluted in saline, paxilline (1 mM) dissolved in dimethyl sulfoxide (Sigma-Aldrich Chemie), saline and dimethyl sulfoxide were injected through a micropipette, drawn from calibrated 1.16-mm internal diameter glass tubing (internal diameter: 30 µm). Injections were made into the vermis close to the recording site using an air pressure system (Picospritzer II, General Valve, Fairfield, NJ), with five pulses of 20 ms. Each pulse delivered a volume of 0.125 µl. Analyses were performed 30 s after the end of the microinjections. No mouse underwent more than one microinjection on the same day and more than a total of two microinjections.

### Tactile Stimulation of the Whisker Region

Facial dermatomes of the whisker regions were stimulated by calibrated air puffs delivered by an air pressure system (Picospritzer), with an air pressure at the source of 2.6 bar. Air puffs (20 ms duration) were applied by a glass pipette (tip diameter 2 mm). The tip was located 1 cm away from the skin of the whisker region at a lateral angle of 50° with respect with the midline of the head. The timing of air puff stimuli reaching the skin area was determined at the beginning of the recording session with a microphone located at the same place as the skin. Electrophysiological response to stimulation was assessed by complex and simple spike analysis. A complex spike firing response was defined as the occurrence of a complex spike during the interval (10–50 ms) following at least 30% of the stimulations. Simple spike firing response was evaluated on a cross-correlogram, including all the stimulations during the recording of a given cell (bin size = 2 ms). The significance of the response was evaluated by comparing the averaged spike number per bin on five successive bins following the stimulation and all the bin values in the interval (−200 to −100 ms) before the stimulation.

In a same experiment, puffs were randomly deliverd each 15 to 60 s. A minimum of 20 puffs were deliverd in each reported experiment.

### Immunofluorescence

Mouse brains were removed and embedded in GSV1 medium (SLEE, Germany) and shock-frozen in liquid nitrogen-cooled 2-methylbutane. Serial 10-µm cryostat sections were performed and transferred to poly-L-lysine-coated glass slides. The slices were washed with Tris-buffered saline (TBS; 150 mM NaCl, 50 mM Tris, pH 7.4) and pre-incubated with 10% normal donkey serum in TBS containing 1% bovine serum albumin and 0.5% Triton X-100 for 4.5 hours. The slices were then washed with TBS and incubated overnight with anti-BKα_(674–1,115)_ and with anti-Somatostatin (Santa Cruz Biotechnology, Santa Cruz, CA) as a molecular marker for Golgi cells [12], [13]. After rinsing with TBS, slices were incubated with an Alexa555-coupled donkey anti-rabbit IgG (Molecular Probes,Carlsbad, CA) for BK channel detection and with an Alexa488-coupled donkey anti-goat IgG (Molecular Probes) for somatostatin detection. After washing with TBS, the sections were cover-slipped in TBS-glycerol (1∶1; pH 8.6) and stored at 4°C protected from light. Immunofluorescence was analyzed with a confocal-laser scanning microscope attached to Nikon Diaphot 300 and equipped with a crypton-argon laser.

### Runway Test

In the runway test, mice were placed on one brightly illuminated extremity of the runway and had to run to the other side to return to their cage. The elevated runway had low obstacles intended to impede their progress. The runway was 100 cm long and 1.2 cm wide. Obstacles consisting of wood rods (1 cm diameter, 1.2 cm width) placed every 10 cm. The numbers of slips of the left hind leg were counted and time spent was noted.

### Data Analysis

All recordings were performed with a bandwidth of 0.01 KHz to 10 KHz. Data was stored on 4-mm digital audio tapes (Sony PCM-R500) and transferred to a Pentium III personal computer with analog-to-digital converter boards (Power 1401, CED, Cambridge, UK). The recorded data were digitized continuously at 10 KHz. Off-line analysis and illustrations were performed using the Spike 2 CED software. The discrimination between PC simple and complex spikes was performed by the same software (waveform recognition) and controlled visually before analysis. Waveform averaging was performed with the corresponding function of this software on a 120 second-minimum recording.

The rhythmic frequency was defined as the reciprocal of the latency of the first peak in the autocorrelogram of simple spike firing (width = 1 s, bin size = 0.2 ms). Consequently, rhythmic frequency could not be determined on flat autocorrelograms. The strength of the rhythmicity was quantified with a rhythm index measured on the simple spike autocorrelogram (120 s-lasting recording, width = 1 s, bin size = 1 ms) [14]. Briefly, the height and depth of all peaks and valleys that were significantly different from the baseline and occurred at specific latencies with regard to the initial peak were summed. The sum was divided by the total number of spikes in the recording. In the autocorrelograms with no significant peaks and valleys, a value of zero was given to the rhythm index and the activity was considered as non-rhythmic. In these cases, or when the rhythm index was less than an empirically determined value of 0.01, the rhythmic frequency was not determined. Thus, we quantified the rhythmicity according to its frequency (by the rhythmic frequency) and its strength by the rhythm index (the higher the rhythmicity, the higher the rhythm index). The regularity of the cell was measured by the coefficient of variation (CV), defined as the quotient between the standard deviation and the mean of the interspike intervals. Local field potential was analyzed using a 4096-point Fast-Fourier-Transform computed from a 15-second recording sample. Oscillation index was obtained by dividing the maximum amplitude of the peak by the area below the curve. In order to test the spatial coherence of local field potential oscillation (LFPO) recordings, cross-correlation functions between each set of two LFPO (α_1_, α_2_) were calculated. The span of time lags or leads was analyzed for a time window (T) corresponding to a recording period of 0.6 s. The cross-correlation function between two functions (e.g., α_1_ and α_2_) was defined as:
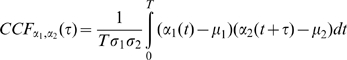
where μ_i_ and σ_i_ are the mean value and the variance of α_i_, and τ is the lag between the two functions. When the signals α_1_(t) and α_2_(t) are statistically correlated, their cross-correlation function displays a peak (a significant cross-correlation function maximum) or a trough (a significant cross-correlation function minimum) at the abscissa τ^*^. Positive values of τ^*^ denote a time lead of α_1_ (t) relative to α_2_ (t), whereas negative values denote a time lag.

We used the Student's t test for unpaired samples to compare WT and mutant mice values. Results are expressed and illustrated as mean±standard deviation (SD) and are considered significant if p<0.05. All statistical analyses were performed using Statistica 6.0.

## Results

### 1. Simple Spike Firing of PCs in BK^−/−^ Is Highly Rhythmic

**Figure 2:**
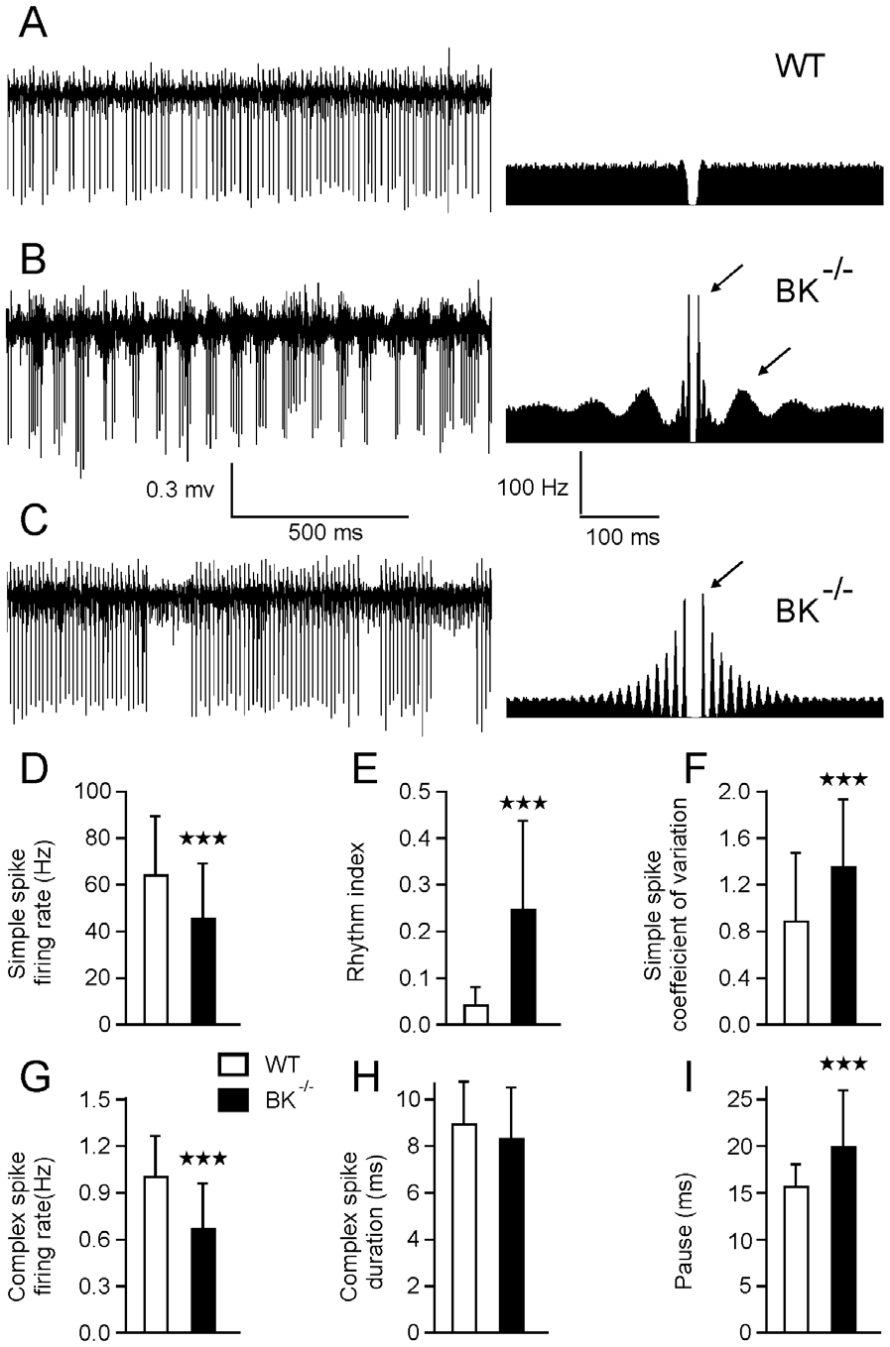
Purkinje cells in BK^−/−^ mice reveal different modes of simple spike firing. (*A*) Representative extracellular recording of a Purkinje cell in a WT (*A*) and a BK^−/−^ (*B–C*) mouse with corresponding autocorrelograms computed on a 120-s sample. Arrows indicate peaks that demonstrate rapid (C) and double rhythmicity (B). Central peak artifacts in the autocorrelograms were deleted, as in the following figures. (*D–I*) Bar graphs of simple spike firing rate (*D*), rhythm index (*E*), CV (*F*), and of complex spike firing rate (*G*), duration (*H*), and pause (*I*) in WT (n = 35) and in BK^−/−^ (n = 48) Purkinje cells. Bars indicate standard deviation. (In this figure and in the following ones, * = p<0.05, , *** = p<0.0001).

A total of 83 PCs (48 in BK^−/−^ and 35 in WT) were recorded and analyzed in 10 mice (5 WT and 5 BK^−/−^) by an investigator blind to the genotype. Fig. 2 illustrates the typical firing pattern (left) and autocorrelogram (right) of PCs recorded in WT mice (Fig. 2*A*) and the two stereotypical firing patterns of PCs recorded in BK^−/−^ mice (Fig. 2*B,C*). Among the 48 PCs recorded in BK^−/−^ mice, 22 (46%) showed a periodic bursting pattern in the beta range (15.2±0.9 Hz) with fast intraburst frequency (117±56 Hz) (Fig. 2*B*). This peculiar mode of firing caused a double rhythmicity on the corresponding autocorrelograms: the rapid rhythmicity corresponds to intraburst firing and the slower rhythmicity corresponds to burst frequency. Thirteen PCs (27%) showed tonic firing with fast rhythmicity (107±64 Hz) (Fig. 2*C*). The remaining 13 cells (27%) showed a similar pattern to the 35 cells recorded in WT animals; these patterns were characterized by a simple spike firing without any consistent rhythmicity, as illustrated by the flat autocorrelogram (Fig. 2*A*, right). These three firing modes could be observed in all BK^−/−^ mice. Occasionally, the switch from one to another mode was observed. Overall, the simple spike firing rate of the PCs recorded in BK^−/−^ was significantly slower than in WT animals (Fig. 2*D*). The mean rhythmic index was significantly greater in BK^−/−^ than in WT mice (Fig. 2*E*). Although PCs recorded in BK^−/−^ were much more rhythmic than those recorded in WT mice, they were also more irregular (mainly because of the presence of bursts), as demonstrated by an increased coefficient of variation (CV) (Fig. 2*F*).

### 2. Pause Following Complex Spikes Is Longer in BK^−/−^ Mice than in WT

PC fire simple and complex spikes. These latter interrupt simple spike firing during a 10–50 ms period, the so-called pause generated complex spike. This pause may be followed by a long-lasting (∼500 ms) inhibition or facilitation of simple spike [15]. Given the presumed function of BK channels in the pause-generated complex spike in simple spike firing, we examined the complex spike firing rate and the subsequent pause in spontaneous PC discharge. We found that complex spike firing rate was significantly lower in BK^−/−^ mice than in controls (Fig. 2*G*). Mean complex spike duration in both groups was similar (Fig. 2*H*), but complex spikes were followed by a longer pause in simple spike firing in BK^−/−^ than in WT mice, which demonstrates that BK channel is not involved in pause generation (Fig. 2*I*).

### 3. BK^−/−^ Mice Present Beta Oscillation in the Cerebellar Cortex

**Figure 3:**
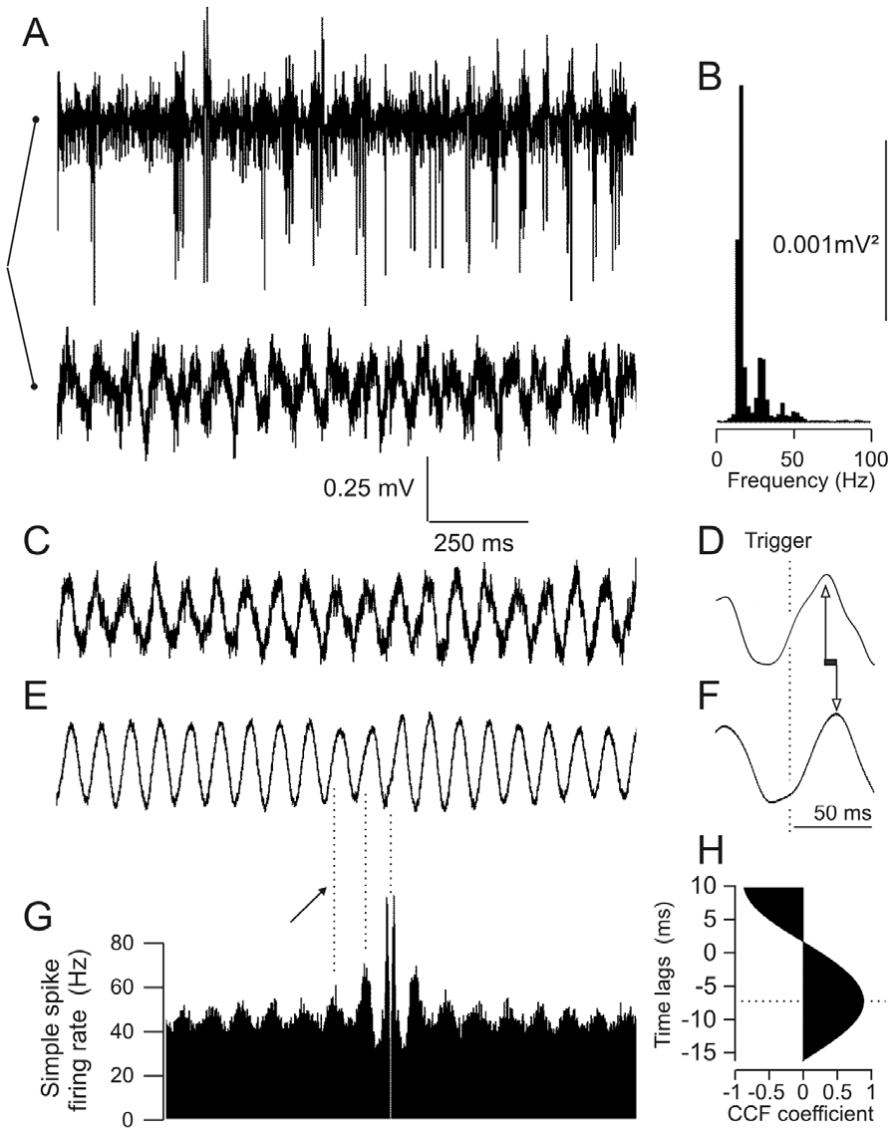
Cerebellar cortices of BK^−/−^ mice present a LFPO in the beta-range phase-locked with both the simple and complex spikes. (*A–B*) Simultaneous recording of a LFPO and a Purkinje cell (250 µm-apart along the parallel fiber axis) and Fast-Fourier-Transform of the LFPO. (*C–F*). Spike-trigger averaging of the LFPO using the complex (C–D) and the simple (E–F) spike. Note the phase-difference in the phase-locking of complex and simple spikes. The smoother aspect of the simple spike triggered wave is due to the much greater number of triggering spikes. Traces D and F are low-pass filtered (<500 Hz); note the difference in time scale. Arrows indicate the time lag. (*G*) Simple spike autocorrelogram of the Purkinje cell illustrated in A. Arrow indicates the correspondence between low frequency rhythmicity and LFPO wave. (*H*) Cross-correlation function between the non-filtered simple and complex spike triggered averaging, confirming the time lag around 7 ms.

We previously demonstrated that mice lacking calcium-binding proteins have increased simple spike rhythmicity and synchronicity that may sustain the emergence of fast LFPO (∼160 Hz) [16]. The frequency of this oscillation was correlated to the rhythmic frequency of PCs [17], [18]. Given the increased rhythmicity of PCs in BK^−/−^ mice, we looked for the presence of LFPO. All BK^−/−^ mice presented beta (12–32 Hz) LFPO in all parts of the explored cerebellum, reaching maximal amplitude close to the PCs layer, whereas none of the WT mice did (Fig. 3*A*, lower trace). This LFPO was constant throughout the recordings. Fourier transform analysis demonstrated a main frequency at 15 Hz and two harmonics peaks at 30 and 45 Hz (Fig. 3*B*). To study the relationships between the PC firing and the LFPO, we simultaneously recorded LFPO and PCs with two distinct electrodes (n = 6). Figure 3*A* illustrates one of these simultaneous recordings. Spike-triggered averaging of the LFPO using the complex (Fig. 3*C,D*) or the simple (Fig. 3*E,F*) spike as trigger revealed the 15 Hz oscillation, demonstrating a tight phase-locking of the LFPO with both complex and simple spikes. In addition, the phase relationship between the simple spike autocorrelogram and the averaged LFPO confirms that the beta oscillation was actually phase-locked with the slow rhythmic frequency of the cells, which also corresponds to burst frequency (Fig. 3*G*). Comparison between Figures 3*D and 3F* illustrates that the relative timing of LFPO and simple or complex spike was different, as the complex spikes occurred at the end of the ascending wave, whereas simple spikes occurred at the beginning of it. To quantify this delay, we cross-correlated simple-spike averaged LFPOs with complex-spike averaged LFPOs in six simultaneous recordings of LFPO and PCs. An example of this analysis is illustrated in Fig. 3*H*. The mean delay between the averaged LFPO triggered by the simple or the complex spike was 10.3±3.9 ms (95% CI = [6.2–14.3]).

To test if non-synaptic transmission is involved in maintaining 12–32 Hz cerebellar LFPO, we tested the effects carbenoxolone (gap junction blocker) on the LFPO frequency and index. We performed five carbenoxolone injections during LFPO recordings, that failed to significantly modify LFPO index (15.9±3.4 Vs 19.1±6.5 p = 0.1) or frequency (25±2 to 24±2 Hz, p>0.2).

### 4. Golgi Cells of BK^−/−^ Mice Are Phase-Locked with the Beta Oscillation

**Figure 4:**
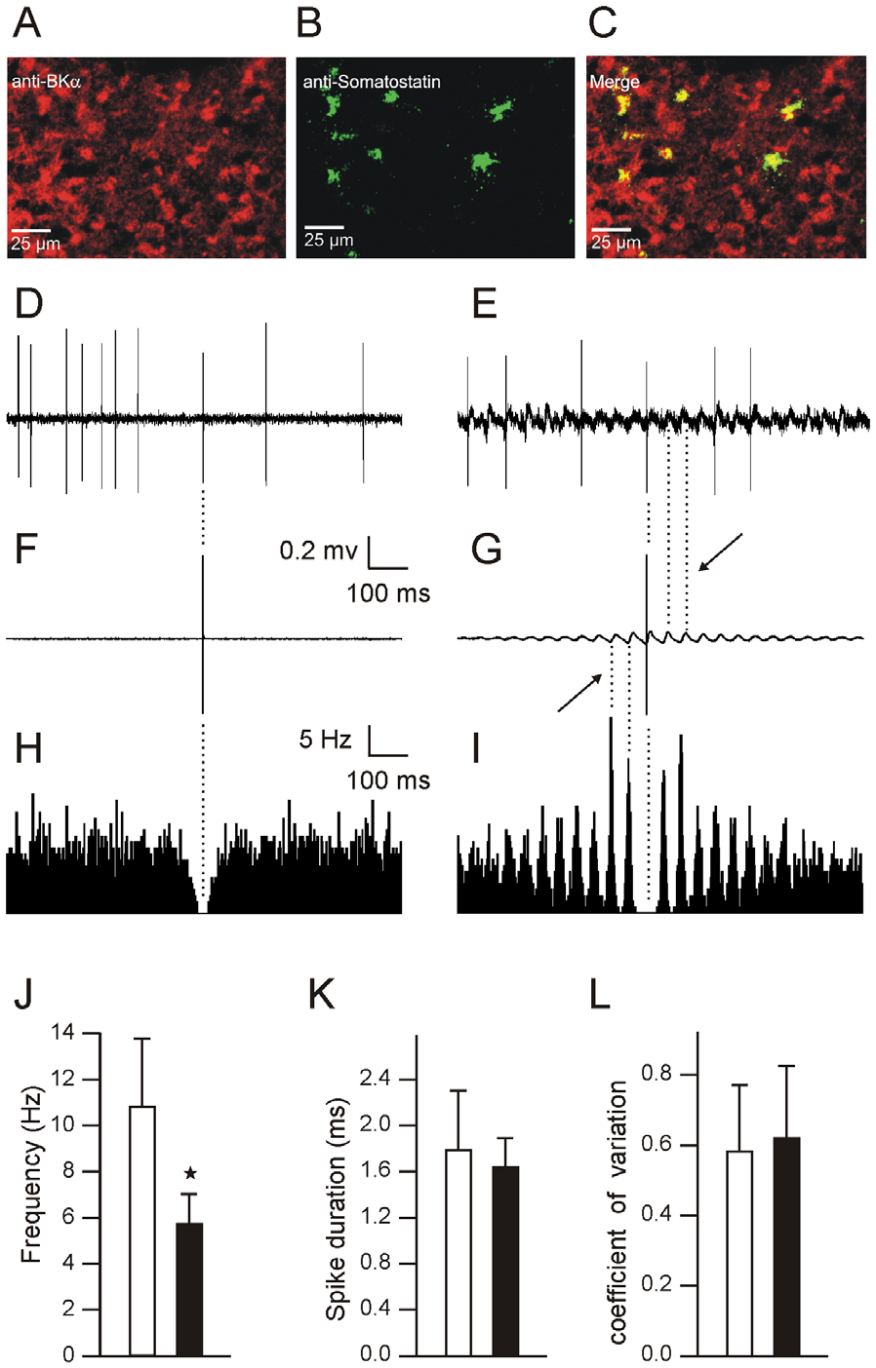
Golgi cells in BK^−/−^ mice exhibit rhythmic firing phase-locked with the LFPO. (*A–C*) Representative confocal laser scan microscopy images of mouse cerebellar cortex granular layer containing numerous and tiny granule cells, a bit larger unipolar brush cells and the much larger Golgi cells. The immunofluorescence of the granular layer shows BK channel expression (A; red) in tiny granule cells and in larger neurons as well as somatostatin expression, a marker for Golgi cells, in a fraction of large neurons (B; green). The colocalization (yellow) demonstrates BK channel-positive Golgi cells (*C*); scale bars: 25 µm. (*D–I*). Extracellular recording of a Golgi cell in a WT (D) and a BK^−/−^ mouse (E) with spike triggered averaging (F–G) and autocorrelograms (H–I) of the corresponding recordings. Note the phase-locking and the rhythmicity of Golgi cells in BK^−/−^ mice. (*J–K*) Bar graphs of Golgi spike frequency (J), duration (K), and CV (L) in WT (n = 5) and in BK^−/−^ mice (n = 5).

Golgi cells are cerebellar neurons formally identified on the basis of spontaneous firing in the alert animal [10]. Immunohistochemistry of the cerebellar cortex revealed that Golgi cells, identified by positive somatostatin staining as a marker [12], [13], express BK channels (Fig. 4*A–C*). To further investigate the relationship between the LFPO and these neurons, we recorded Golgi cells in five WT mice and in five BK^−/−^ mice. Typical recordings of the spontaneous firing of a Golgi cell in a WT and in a BK^−/−^ mouse are shown in Fig. 4*D and 4E*, respectively. The spike-triggered averaged trace of the recording demonstrated a non-oscillating background activity in the WT (Fig. 4*F*). In the BK^−/−^ (Fig. 4*G*), the same procedure revealed a 26 Hz oscillation before and after the Golgi spike, demonstrating that this spike was phase-locked with the LFPO. All Golgi cells recorded simultaneously with a LFPO demonstrated a similar phase-locking of their spikes. Their autocorrelograms were characterized by a high level of rhythmicity, as illustrated in Fig. 4*I*, whereas such rhythmicity was never observed in WT mice (Fig. 4*H*). As illustrated in Fig. 4*G and 4I*, the rhythmic frequency of Golgi cell spikes corresponded to the frequency of LFPO. Although phase-locked with a >10 Hz oscillation, the firing rates of Golgi cells in BK^−/−^ mice were significantly lower than in WT (Fig. 4*J*). However, there were no significant differences in Golgi spike duration (Fig. 4*K*) or CV (Fig. 4*L*) between BK^−/−^ and WT mice.

### 5. Beta Oscillation Is Synchronized along Both Frontal and Sagittal Planes

**Figure 5:**
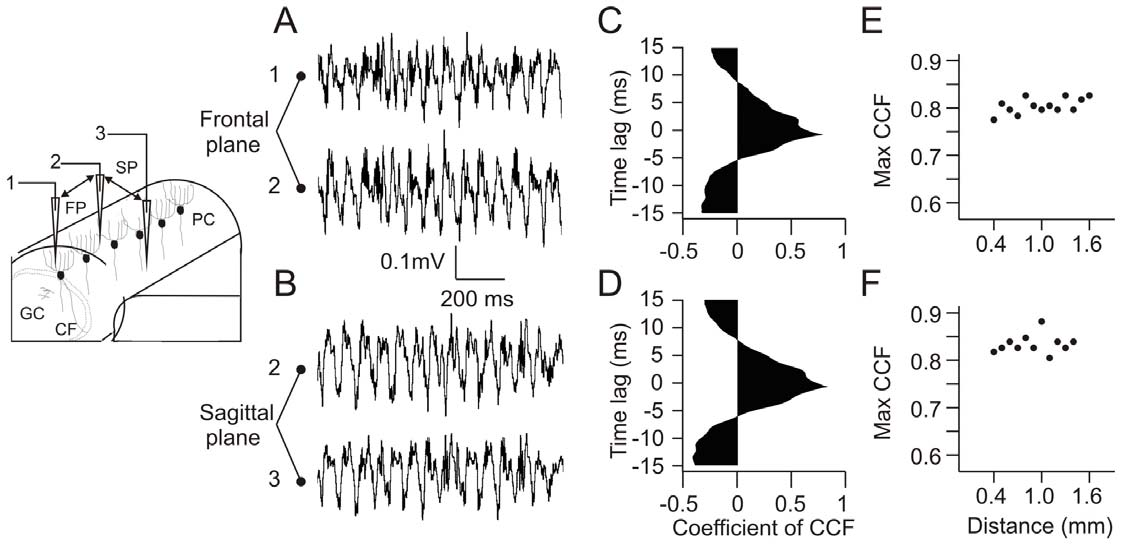
LFPO in BK^−/−^ mice is highly synchronized along the frontal and sagittal plane. (*A–B*) Simultaneous recordings of two LFPO with electrodes at a distance of 400 µm apart along the frontal (A) and sagittal (B) planes. (*C–D*) Cross-correlation function (CCF) of the recorded signals illustrated in A and B. (*E–F*) Plotted values of the maximal CCF coefficient and the corresponding distance between recording electrodes in a same BK^−/−^ mouse in the frontal (E) and in the sagittal (F) plane. Note the absence of significant variation.

We recorded 19 pairs of LFPOs separated by 0.25 to 1.8 mm along the frontal plane and 16 pairs separated by 0.4 to 1.5 mm along the sagittal plane. Cross-correlation functions demonstrated a tight correlation between the unfiltered recorded signals along the frontal (0.81±0.02) and the sagittal (0.83±0.03) planes. We did not find any significant relationship between the time lag or cross-correlation coefficient and the distance between electrodes. One of these experiments is illustrated in Fig. 5; in this case, microelectrode #2 that records a LFPO was left in place while the two other microelectrodes were successively displaced along the sagittal (#3) and the frontal planes (#1). Simultaneous recordings of LFPO in these planes (Fig. 5*A,B*) were cross-correlated (Fig. 5*C,D*), demonstrating highly significant maximal cross-correlation coefficients independent of the interelectrode distance (Fig. 5*E,F*). Moreover, time lags remained constant, close to 0 ms, whatever the interelectrode distance.

### 6. PCs in BK^−/−^ Mice Reveal a Shorter Inhibitory Response to Tactile Stimulation

**Figure 6:**
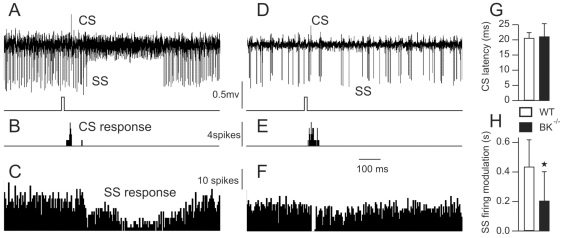
Simple spike response of Purkinje cells to tactile stimulation is altered in BK^−/−^ mice. (*A*) Purkinje cells recorded in the Crus2A of a WT mouse during the stimulation of the whisker region, timing of stimulation is illustrated in the lower trace (CS = complex spike, SS = simple spike). (*B*,*C*) Bar graphs of complex (B) and simple (C) spike firing, 15 trials summed. (*D*) Purkinje cells recorded in the Crus2A of a BK^−/−^ mouse during the stimulation of the whisker region. (*E*,*F*) Bar graph of complex (E) and simple (F) spike firing, 26 trials summed. (*G*) Bar graph of timing between stimulus and complex spike firing in WT and BK^−/−^ mice. (*H*) Bar graph of simple spike response duration in WT and BK^−/−^ mice.

Synchronization in the frontal and sagittal planes, highly rhythmic firing pattern of Purkinje and Golgi cells, and the phase-locking of the latter with the LFPO suggest a profound functional disorganization of the cerebellar cortex in BK^−/−^ mice. PCs appear to be ‘trapped’ in stereotyped, non-adaptable firing. To test this hypothesis, we recorded 22 PCs in Crus 2A (where project trigeminal inputs in rodents [19]) of WT (n = 7) and BK^−/−^ (n = 15) mice and studied their responses to stimulation of the whisker region. To be considered for analysis, a PC had to present a response in the simple and/or in the complex spike firing pattern. A representative recording of a Crus 2A PC in a WT mouse during the stimulation of the whisker region dermatome is illustrated in Fig. 6*A*. Around 20 ms after each stimulation, a complex spike was fired (Fig. 6*B*), followed by a prolonged inhibition (∼500 ms) of simple spike firing (Fig. 6*C*). An analogous experiment performed in BK^−/−^ mice (Fig. 6*D*) demonstrated a similar response in complex spike firing (Fig. 6*E*), but much shorter inhibition in simple spike firing (Fig. 6*F*). Statistical analysis confirmed the absence of a significant difference in the latency of complex spike response (Fig. 6*G*) and the shorter duration in simple spike inhibition of BK^−/−^ PCs relative to WT (Fig. 6*H*).

### 7. Injection of Paxilline Mimics BK^−/−^ Firing Pattern in Wild-Type Mice

**Figure 7:**
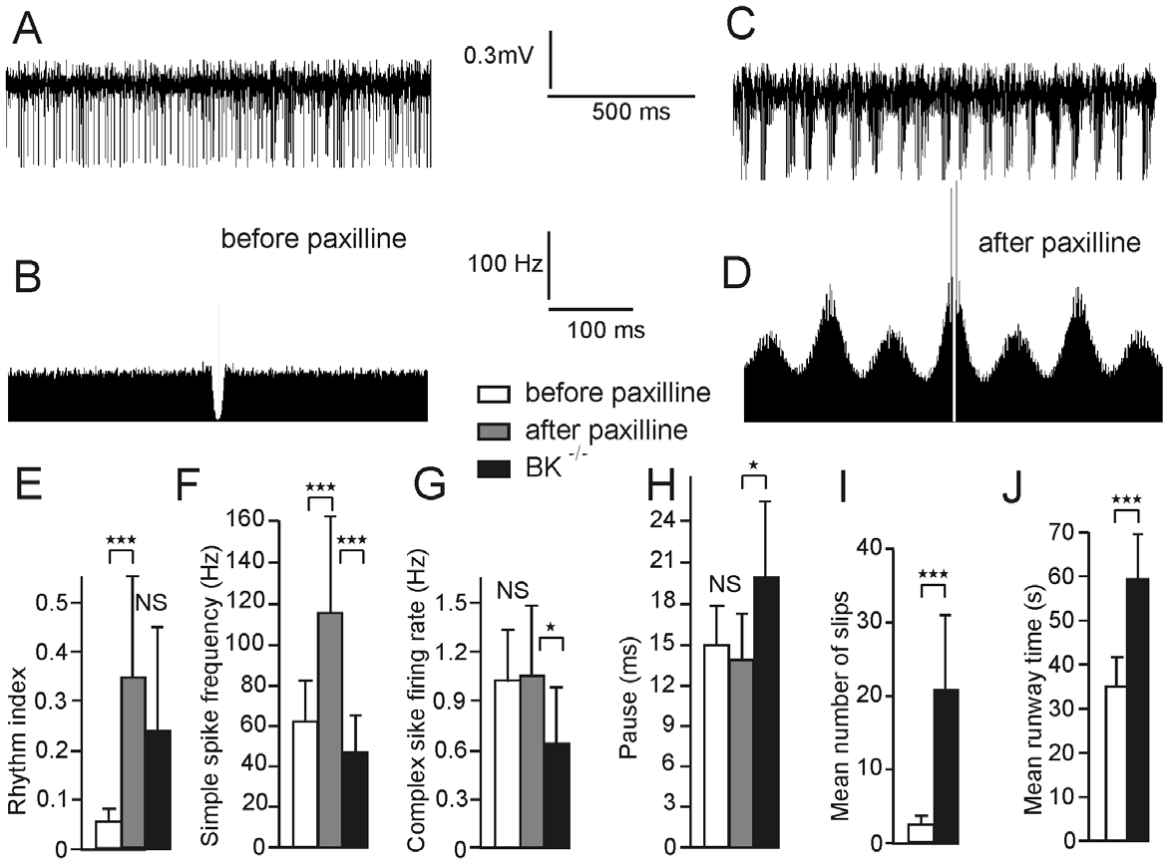
Intracerebellar microinjection of paxilline in WT mice reproduces the rhythmic firing of Purkinje cells and the ataxic behavior of BK^−/−^ mice. (*A*,*B*) Spontaneous firing of a Purkinje cell recorded in a WT mouse (*A*) and corresponding autocorrelogram (*B*). Note the absence of rhythmicity. (*C*,*D*) The same, following microinjection of paxilline. (*E–H*) Bar graphs of Purkinje cells simple spike rhythmicity (n = 13)(*E*) and frequency (n = 13)(*F*), Purkinje cells complex spike frequency (n = 8)(*G*) and subsequent pause duration (n = 8) (*H*) before and after paxilline injection and in BK^−/−^ (n = 48, value illustrated in fig 2 and reproduced here for comparison purpose). Stars indicate significance as in fig 2, for student t test for paired values (comparison between before and after injection) and unpaired values (comparison between WT PC after injection and PC in BK^−/−^) (*I*,*J*) Runway test, bar graph of mean number of slips (*I*) and time to reach the end of the bar (*J*) before and after paxilline injection (n = 9).

The highly rhythmic firing pattern of PCs of BK^−/−^ mice may be due to the absence of the normal control of BK channel on PC rhythmicity. To test this hypothesis, we microinjected paxilline, a BK channel blocker, into the vermis of WT mice close to the recording site of PC. Fig. 7(*A–D*) illustrates recordings made before and after microinjection of paxilline. Before the injection, the cell was poorly rhythmic as demonstrated by the flat autocorrelogram (Fig. 7*A,B*). After the injection, the cell became highly rhythmic and the firing frequency tended to increase (Fig. 7*C,D*). The same experiment, reproduced in thirteen WT mice, demonstrated a dramatic increase in rhythmicity following paxilline microinjection (Fig. 7*E*), mimicking the firing pattern of BK^−/−^ mice illustrated in Fig. 2*B*, and a moderate increase in spike frequency (Fig. 7*F*). This highly regular bursting pattern was accompanied by LFPO at 21.0±4.2 Hz; this LFPO was never observed in untreated WT mice, or in WT mice following dimethyl sulfoxide microinjection. No significant differences were observed in complex spike firing rate (Fig. 7*G*) and subsequent pause (Fig. 7*H*) before and after paxilline injection in eight PC.

In comparison with PC recorded in BK^−/−^ mice, PC recorded in WT following paxilline injection presented a similar increase in rhythm index (Fig. 7*E*), but an increase rather than an increase in simple spike frequency, and no difference in complex spike firing rate and duration (Fig. 7*G,H*).

In order to rule out that paxilline acts through a non BK channel related mechanism, simple spike frequency and rythmicity was studied during paxilline injections in BK^−/−^ mice. Paxilline was injected during the recording of 9 PC that presented a rhythm index below 0.25, in order to keep the possibility to increase it. Rhythm index was not significantly different after (0.14±0.05) and before the injection (0.15±0.05, p>0.7). Similarly, simple spike firing rate was not different after (58.0±36.0 Hz) and before (57.0±37.0 Hz, p>0.7) injection.

### 8. Intracerebellar Injection of Paxilline Causes Ataxia in Wild-Type Mice

To test if BK channel inactivation was related to the ataxia observed in BK^−/−^ mice, we tested motor coordination in WT mice by the classical runway test before and after microinjection of paxilline into the vermis. The effects of this BK channel blocker were highly reproducible and characterized by a significant increase in the number of slips (Fig. 7*I*) and the time spent for walking on the bar (Fig. 7*J*) in 9 WT mice. The walking movements of the mice were severely altered after treatment with the BK channel blocker; mice frequently slipped on both fore- and hind-limbs, resulting in loss of balance on the bar. The ataxia induced by paxilline injection was less severe than in BK^−/−^. In these mice, runway test could not be performed since the BK^−/−^ mice fell off the bar quickly after that there were placed on it. In contrast, microinjection of the same volume of dimethyl sulfoxide did not induce ataxia in WT mice (n = 3).

## Discussion

We have demonstrated that BK channels tightly control the firing rhythmicity of Purkinje and Golgi cells. Inactivation of BK channels in mice, either by targeted deletion or pharmacological blockade, locks Purkinje and Golgi cells in highly rhythmic and synchronous firing, which possibly results in motor impairment. *In vitro*, PCs have a tendency to fire in bursts [2], [3], [20]–[22]. This indicates that intrinsic ionic conductance is responsible for burst generation. In contrast, in awake WT animals, this firing mode is relatively rare. We show here that it represents the dominant firing mode in BK^−/−^ PCs or during pharmacological blockade of BK channels in WT mice. This suggests that BK channels maintain PCs in a non-bursting mode *in vivo*. *In vitro*, T-type Ca^2+^-channels and the resurgent Na^+^ currents make the major contribution to membrane depolarization. These inward currents are opposed by the Ca^2+^-activated, potassium BK current. A cumulative inactivation of Na^+^-channels and a cumulative increase in SK current and dendritic Ca^2+^ spikes terminate the burst. Except for P/Q-type Ca^2+^-channels required for dendritic Ca^2+^ spike [23], no burst modulating channels are necessary to sustain regular spontaneous bursting [21]. Blockade of BK channels decreases burst duration in slices, suggesting that these channels slow down the depolarizing phase of the burst [21]. Given the role of P/Q Ca^2+^ channels in the generation and control of burst firing in PCs, the emergence of spontaneous bursting sustained by beta LFPO in BK^−/−^ PCs and after BK blockade in WT mice suggests that these channels might be involved in maintaining the non-bursting mode. In BK^−/−^ mice, 27% of PC present a normal mode of firing during the recording, which demonstrates that BK channel inactivation does not lock the cells in a bursting or in a rhythmic mode of firing, but rather facilitates the cells to maintain or switch to these modes. The difference between PC in BK^−/−^ cells and PC in WT mice following paxilline injection in terms of simple spike firing rate, pause duration or percentage of cells in bursting mode may be due to the different mechanisms of BK channel inactivation. After genetic knocking out, PC may compensate for the absence of BK channel by over- or under-expressing other proteins, which is not expected to occur within seconds after pharmacological inhibition of BK channels in WT.

### Deletion of BK Channels Does Not Alter Simple Spike Pause

The pause in simple spike firing that follows a complex spike is likely to be an important signal for the DCN by producing at this level a transient relief of the inhibition followed by excitatory rebound. Although the underlying mechanism of the pause is poorly understood, Davie et al [24] recently demonstrated that the climbing fiber-evoked dentritic spikes of the PC regulate the duration of the pause. The Ca^2+^influx triggered by the climbing fiber at the dendritic level activates Ca^2+^-dependent potassium conductance [2], [25], inducing hyperpolarization and the related pause in simple spike firing. Small (SK) and large (BK) conductance Ca^2+^-activated potassium channels contribute to the action potential shape and spike after-hyperpolarization, respectively [2]. Our results rule out the hypothesis of a role of BK channels in generating the pause, as we found the pause to be longer in BK^−/−^ than in WT PCs, and unchanged following paxilline microinjection.

### Comparison between Beta and Other Cerebellar Oscillations

Different oscillations have been described *in vivo* in the cerebellum [26], [27], [for a review], [see 28,29]. Oscillations in the beta range have been recorded in quietly sitting normal animals and are inhibited by passive or active movements [26]. This local field potential generated in the granular layer is involved in movement preparation and the PC firing is mainly phase-locked to this rhythm just before movement execution [30]. In a view of the cerebellum as a phase–modulating device [31] such phase relationship between beta oscillation, PC firing and movement execution may be crucial. In the BK^−/−^ mice and during the pharmacological blockade of the BK channels in WT mice, continuous beta oscillation disrupts the timing function of the cerebellar cortex. We hypothesize that this disruption may be the cause of the severe ataxia.

In hippocampus, pharmacological blockade of BK channel also lead to altered function [32], but in vivo recordings demonstrate increased pyramidal cells firing.

New experiments in behaving animals are needed to test the possibility that such oscillation represents an attempt by the cerebellum to compensate for the primary (genetically or pharmacologically induced) functional deficit.

Fast (160 Hz) LFPO related to synchronous and rhythmic firing patterns of PCs has only been documented in ataxic mice [16], [17], [33]–[35], but the ataxia observed in those mutants is not as pronounced as in BK^−/−^ mice. Ataxia is observationally evident in free walking BK^−/−^ mice. In addition to differences in LFPO frequency, there are at least three major differences between fast (∼160 Hz) and beta (12–32 Hz) cerebellar oscillation. First, beta oscillation is synchronized along both parallel and frontal axes, whereas fast oscillation is synchronized only along parallel fibers axis. Secondly, beta oscillation is not altered by gap junction blocker, whereas fast oscillation is. Thirdly, Golgi cells are phase-locked with beta but not with 160 Hz oscillation.

### BK Channel Deletion Decreases Simple-Spike Delayed Inhibition

BK^−/−^ mice PCs were responsive to peripheral stimulation by a conserved climbing fiber input but simple spike firing modulation (expressed by delayed inhibition) was severely altered. This could be due to entrainment of simple spike firing into the stereotyped bursting. However, although complex spike firing was also trapped in the beta oscillation, it remained normally responsive to peripheral stimulation. Another explanation could be that BK channels are directly involved in the simple-spike delayed inhibition. This putative inhibitory effect might be triggered by Ca^2+^ input into the PCs related to climbing fiber activation. Preservation of the pause duration argues against the involvement of BK-control in delayed after-hyperpolarization.

### BK Channel Deletion Changes Golgi Cells Rhythmicity

Golgi cells exert a time-windowing effect on the temporal dynamics of granule cells response through their inhibitory control [31]. They could also reflect a climbing fiber-dependent modulation throughout sagittal arrays of granules cells [36]. Therefore, loss of Golgi cell BK channels and the fact that their firing discharges were continuously phase-locked to beta oscillation in BK^−/−^ mice suggest that this rhythmic alteration could disrupt their inhibitory control on granule cell output, also contributing to abnormal PC firing and related ataxia. Selective ablation of Golgi cells causes severe ataxia [37], [38]. The shaping of mossy fibers-induced NMDA depolarization by Golgi-GABA inhibition is important for the temporal summation of mossy fiber input [38]. Moreover, the absence of BK channels in the Golgi cells may interfere with the cascade of events that follow their AMPA-mediated excitation by mossy fibers. Notably, the Ca^2+^ influx stimulates BK channels, contributing to fast after-hyperpolarization [39]. The inhibitory output of PCs is also able to modulate Golgi cells action, therefore possibly inducing phase-locking to beta oscillation.

### From Abnormal Rhythmicity and Synchronicity of PC Simple Spike Firing to Ataxia

Firing modulation of the neurons of the deep cerebellar nuclei by the PCs is responsible for movement coordination [40]. In this context, the emergence of stereotyped bursting of the PCs phase-locked to beta LFPO may correspond to a new type of pathophysiological mechanism of cerebellar ataxia. The high frequency burst of inhibitory postsynaptic potentials induced by PC axon stimulation produces rebound depolarization and hyperexcitabilty of deep cerebellar neurons [41].

Physiological simple spikes are poorly rhythmic and non-synchronous [16]–[18], [28], [34], [35], [42], [43]. The lower simple spike firing rate in BK^−/−^ PCs, or the lower complex spike firing rate and longer subsequent pause do not account for the severe ataxic phenotype of mutants, since similar abnormalities in simple and complex spike firing have been described in other mouse models with mild or absent motor coordination impairment [43]. Moreover, it was not observed following paxilline microinjection in WT. Actually, the alteration in PC firing pattern is much more striking than the firing rate changes in BK^−/−^ mice and could be reproduced by microinjection of paxilline into the vermis of WT mice, which also produced ataxia. Simple spike rhythmicity is increased in many models of ataxic animals [16], [17], [33]–[35], [44], whereas WT mice PCs present little if any rhythmicity. The optimal functional state of PC population seems to be situated between excessive irregularities leading to ataxia [45], [46] and persistent rhythmicity, also resulting in ataxia. The study of Medina and Lisberger [47] demonstrates that the optimal control of eye movement pursuit exerted by a PC population corresponds to transition from highly covariant simple spike firing during movement initiation to more independent firing later on. This emphasizes the importance of balance between synchrony and asynchrony for the dynamic modulation of the cerebellar output [48].

The induction of ataxia by BK blockers in the cerebellar cortex demonstrates that one of the mechanisms leading to ataxia involves the cerebellar cortex. However, the contribution of other cerebellar cells such as granule cells, which also express BK channels [49] or the implication of other regulatory mechanisms cannot be ruled out by the present study. Granule cell electrophysiological activity has been documented *in vivo* in anesthetized mice [36] and in decerebrated cats [50] where they are considered as signal-to-noise enhancing threshold elements. However, single recording of granule cells in alert mice has not been described to our knowledge. Recordings of PC in PC-specific BK^−/−^ mice could help to discriminate what is specific to PC in the behavior and in the cerebellar electrophysiology.

Our results provide direct evidence that BK channels play a crucial role not only in the intrinsic properties of the different neurons but also on the network properties that influence the final input-output transformation of the cerebellar cortex. We propose that the emergence of beta oscillation and the temporal coding modification of the spike firing of both Purkinje and Golgi cells are among the main causes for the induced ataxia observed in BK^−/−^ mice. In this view, one may suppose that beta oscillation in the BK^−/−^ interfere with the normal beta oscillation in the granular layer controlled by the Golgi cells in normal animals [24].
